# Cerium oxide nanoparticles improve liver regeneration after acetaminophen-induced liver injury and partial hepatectomy in rats

**DOI:** 10.1186/s12951-019-0544-5

**Published:** 2019-10-31

**Authors:** Bernat Córdoba-Jover, Altamira Arce-Cerezo, Jordi Ribera, Montse Pauta, Denise Oró, Gregori Casals, Guillermo Fernández-Varo, Eudald Casals, Victor Puntes, Wladimiro Jiménez, Manuel Morales-Ruiz

**Affiliations:** 1 Biochemistry and Molecular Genetics Department, Hospital Clínic of Barcelona, IDIBAPS, CIBERehd, 170 Villarroel St., 08036 Barcelona, Spain; 2 Working Group for the Biochemical Assessment of Hepatic Disease-SEQC-ML, Barcelona, Spain; 3 Vall d’Hebron Institut of Research (VHIR), Barcelona, Spain; 4 Institut Català de Nanociència i Nanotecnologia (ICN2), CSIC and The Barcelona Institute of Science and Technology (BIST), Barcelona, Spain; 5 Institució Catalana de Recerca i Estudis Avançats (ICREA), Barcelona, Spain; 6 School of Biotechnology and Health Sciences, Wuyi University, Jiangmen, 529020 China; 7 Department of Biomedicine-Biochemistry Unit, School of Medicine, University of Barcelona, Barcelona, Spain

**Keywords:** Liver regeneration, Oxidative stress, Cerium oxide nanoparticles, Partial hepatectomy, Acetaminophen-induced liver injury

## Abstract

**Background and aims:**

Cerium oxide nanoparticles are effective scavengers of reactive oxygen species and have been proposed as a treatment for oxidative stress-related diseases. Consequently, we aimed to investigate the effect of these nanoparticles on hepatic regeneration after liver injury by partial hepatectomy and acetaminophen overdose.

**Methods:**

All the in vitro experiments were performed in HepG2 cells. For the acetaminophen and partial hepatectomy experimental models, male Wistar rats were divided into three groups: (1) nanoparticles group, which received 0.1 mg/kg cerium nanoparticles i.v. twice a week for 2 weeks before 1 g/kg acetaminophen treatment, (2) *N*-acetyl-cysteine group, which received 300 mg/kg of *N*-acetyl-cysteine i.p. 1 h after APAP treatment and (3) partial hepatectomy group, which received the same nanoparticles treatment before partial hepatectomy. Each group was matched with vehicle-controlled rats.

**Results:**

In the partial hepatectomy model, rats treated with cerium oxide nanoparticles showed a significant increase in liver regeneration, compared with control rats. In the acetaminophen experimental model, nanoparticles and *N*-acetyl-cysteine treatments decreased early liver damage in hepatic tissue. However, only the effect of cerium oxide nanoparticles was associated with a significant increment in hepatocellular proliferation. This treatment also reduced stress markers and increased cell cycle progression in hepatocytes and the activation of the transcription factor NF-κB in vitro and in vivo.

**Conclusions:**

Our results demonstrate that the nanomaterial cerium oxide, besides their known antioxidant capacities, can enhance hepatocellular proliferation in experimental models of liver regeneration and drug-induced hepatotoxicity.

## Background

The liver regenerative capability is essential in the success of some treatments for chronic liver diseases, such as tumor resection and donor liver transplantation, which are conditioned by adequate liver regeneration [1, 2]. However, there are some clinical situations in which the liver shows poor regenerative capacity, such as in the case of liver cirrhosis and severe drug-induced liver injury (DILI) [3, 4].

One pathological process that is common to these two clinical conditions is oxidative stress, which is caused by the excessive formation of reactive oxygen species (ROS). The presence of oxidative stress has been described in most of the clinical conditions associated with chronic liver injury (i.e.: nonalcoholic steatohepatitis, hepatitis C viral infection, alcoholic liver cirrhosis) [5, 6]. In addition, recent reports suggest that drug-induced oxidative stress also significantly correlate with increased DILI risk [7]. Concurrently, the association between high levels of oxidative stress and a reduction of antioxidant defenses has also been reported in most of these pathological situations [8, 9]. According to the results obtained by several studies, the imbalance between the production of ROS and antioxidant defense in some of these liver diseases affects liver regeneration [10–12]. One of the reasons that explain this ROS effect is that ROS modulates the expression of a variety of regulators that play major roles in liver regeneration, including growth factors, transcription factors and cell cycle proteins such as β-catenin [13], cyclin D [14], p53 [15], Nrf2 [16] and JNK/p38 mitogen activated kinases [17].

Cerium oxide nanoparticles (CeO_2_NPs) have drawn considerable attention as a potential therapeutic tool in the prevention and treatment of oxidative stress related diseases. This interest relies on the multi-enzyme mimetic properties of CeO_2_NPs due to their unique electronic structure [18–20]. At the nanoscale, the oxygen vacancies that appear in the CeO_2_ nanocrystals modify their electronic structure enabling them to participate as a catalyst in a wide range of reactions (e.g. promoting the simultaneous oxidation of Carbon Monoxide (CO) and hydrocarbons to CO_2_ and the reduction of Nitrogen Oxides (NOx) to N2 in three way catalytic converters) [21]. Similarly, in biological contexts, it has been reported that cerium oxide nanoparticles can mimic enzymatic antioxidants such as superoxide dismutase [22] and catalase [23]. The beneficial effects of CeO_2_NPs have been reported in different clinical conditions associated with excess production of ROS such as neurology [24–27], diabetes [28, 29], chronic inflammation [30], cirrhosis [31] and cancer [32–34].

Considering the above, we hypothesize that CeO_2_NPs may improve liver regeneration by scavenging ROS in regenerative livers. To test our hypothesis we studied the hepatic regenerative process using two different rat experimental models of liver regeneration that are commonly associated with ROS production: partial hepatectomy (PHx) [35, 36] and DILI caused by acetaminophen (APAP) overdose. In the last case, the therapeutic effect of CeO_2_NPs was compared with the gold standard treatment for APAP-induced injury, *N*-acetylcysteine (NAC) [4].

## Results

### Characterization and biodistribution of cerium oxide nanoparticles in rats

**Fig. 1:**
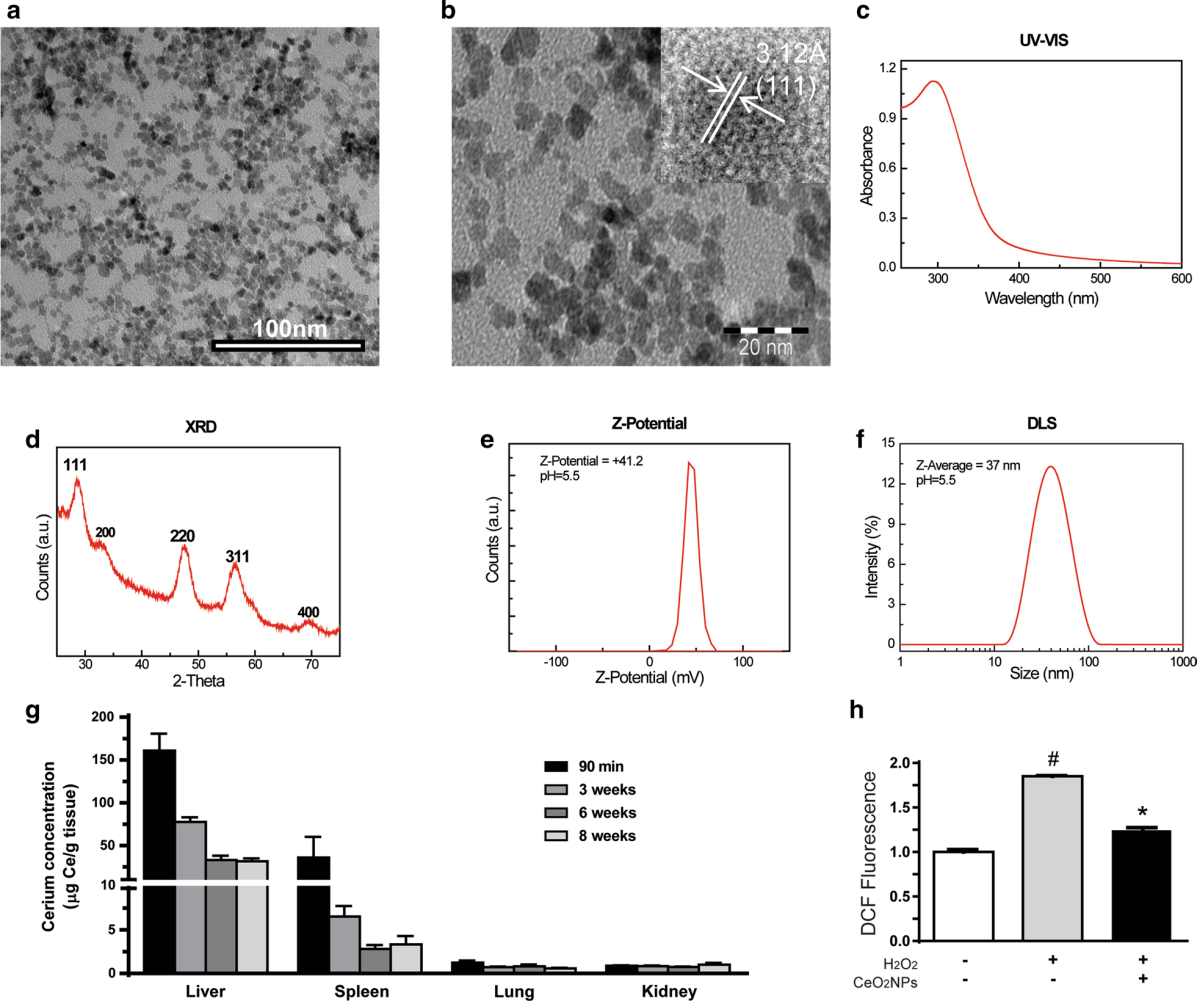
Characterization of cerium oxide nanoparticles. **a**, **b** Representative TEM images of CeO_2_NPs at different magnifications showing the non-aggregate and spherical shape of the engineered nanoparticles. Inset in **b** is a High Resolution TEM image of single particle showing pure CeO_2_ atomic planes; **c** UV–Visible absorption spectrum of the as-synthesized CeO_2_NPs; **d** XRD spectrum of the as-synthesized CeO_2_NPs after being dried under vacuum. **e** Z-Potential distribution and **f** Hydrodynamic diameter measured by DLS of the CeO_2_NPs dispersed in the physiological media (saline solution at pH = 5.5); **g** Cerium concentration in liver, spleen, lung and and kidney from rats treated with CeO_2_NPs for 90 min, 3, 6 and 8 weeks (n = 4 for each group). **h** Oxidative stress was quantified in non-treated and CeO_2_NPs-treated HepG2 cells by measuring DCF fluorescence in basal condition and after inducing oxidative stress with 2 mM H_2_O_2_ added to the culture medium (^*#*^*p *< 0.0001 *vs* basal and *p < 0.0001 vs. non-treated, n = 10)

Cerium oxide nanoparticles (CeO_2_NPs) were analyzed by HR-TEM. The engineered nanoparticles showed spherical morphology (Fig. 1a, b) and were predominantly in the size range of 4 nm. The UV–visible absorption spectrum of CeO_2_NPs showed a characteristic absorption peak of Ce^4+^ at 298 nm (Fig. 1c). The X-ray diffraction pattern of the CeO_2_NPs showed pure CeO_2_NPs with the typical peak broadening characteristic of nanosize particles (Fig. 1d). Measured zeta potentials of the CeO_2_NPs were (+) 41.2 mV (Fig. 1e). The hydrodynamic diameter of the CeO_2_NPs dispersed in saline solution at pH = 5.5 was 37 nm (Fig. 1f). These optimally engineered nanoceria were used further in our animal studies.

Several studies have described that after systemic distribution small inorganic NPs accumulate in the liver and spleen [31, 37]. In agreement, CeO_2_NPs treated rats showed CeO_2_NPs retention into the liver and, to a lesser extent, in spleen as early as 90 min following i.v. injection. In these organs and at this time point, CeO_2_NPs reached concentrations of 160.9 μg and 36.0 μg of CeO_2_NPs per gram of tissue, respectively (Fig. 1g). Interestingly, cerium was still detected in liver and spleen for over 8 weeks although in lower concentrations. CeO_2_NPs retention was barely detected in the lungs and the kidneys of the rats at different time points after the intravenous injection (Fig. 1g).

To investigate the antioxidant properties of CeO_2_NPs, we induced oxidative stress in the hepatocyte cell line HepG2 by H_2_O_2_ treatment, as previously reported [38, 39]. ROS were assessed in these cells by using the dichlorofluorescein (DCF) assay [31]. When exposed to H_2_O_2_, CeO_2_NPs-treated HepG2 cells showed a significant reduction in the accumulation of DCF in comparison to that observed in non-treated cells (Fig. 1h).

### Rats treated with CeO_2_NPs showed increased liver regeneration and hepatocellular proliferation after PHx

**Fig. 2:**
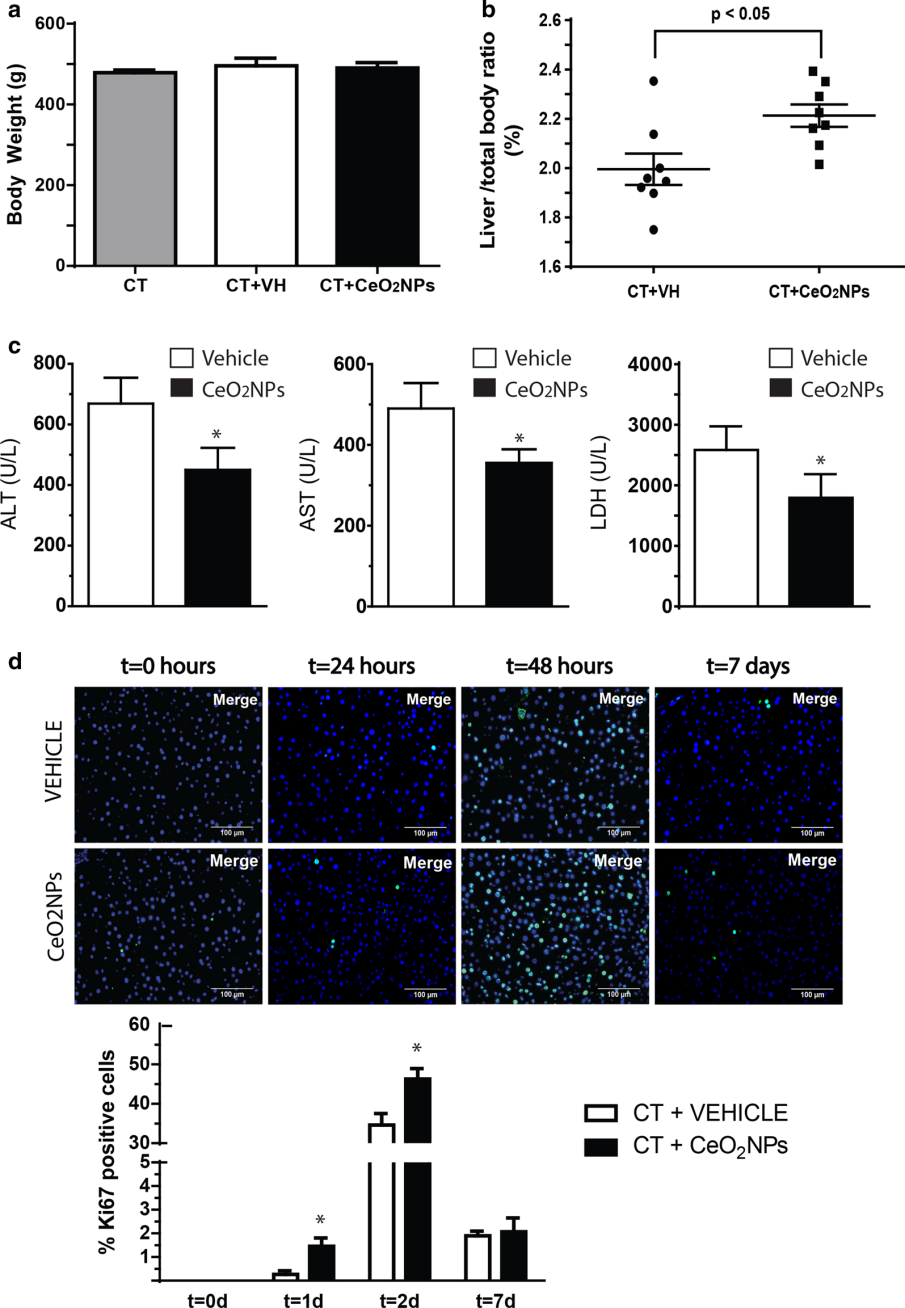
CeO_2_NPs treatment increased liver regeneration and cell proliferation after PHx. **a** Body weights of control rats without treatment and rats that received vehicle or CeO_2_NPs before PHx (n = 8). **b** Hepatic regenerative index at day 6 after PHx (n = 8, *p *< 0.05). **c** Blood levels of ALT (*p < 0.01), AST (*p < 0.05) and LDH (*p < 0.05) in vehicle or CeO_2_NPs-treated rats after 3 h post-PHx (n = 8; mean ± SEM). **d** Representative immunostaining for the Ki-67 antigen in liver histological sections of rats treated with either vehicle or CeO_2_NPs at different time points (t = 0 h, 24 h, 48 h, 7 days). Merged images show co-localization of Ki-67 (green) and nuclear DNA (DAPI, blue). Original magnification ×200 (n = 8 for each group and treatment). On the bottom, percentage quantification of positive Ki-67 liver cells for each time point and treatment (n = 8; mean ± SEM; *p < 0.05 compared with vehicle at the same time points)

Oxidative stress mediate cell growth arrest and impairs hepatic regeneration in mice [13, 14]. Therefore, testing new anti-oxidant drugs to improve liver regeneration has clinical interest. To this aim, we studied the effect of CeO_2_NPs treatment on liver regeneration after performing PHx in rats. Rats were treated with 0.1 mg/kg CeO_2_NPs intravenously twice a week for 2 weeks before PHx. As shown in Fig. 2a, we did not observe any substantial change in body weight between the groups without treatment, vehicle treatment, and CeO_2_NPs treatment. Also, we performed liver laboratory tests in rat serum to quantify the liver function (glucose and albumin) and the liver damage (ALT and AST) in response to the CeO_2_NPs treatment in rats that were fasted for 12 h. We did not detect any significant change of these laboratory parameters between vehicle and CeO_2_NPs treated groups (Additional file 1: Figure S1). These results support the concept that the CeO_2_NPs pretreatment is safe for the liver and is not associated with detectable side effects in the short term. Rats were sacrificed 6 days after the surgical procedure and the wet liver remnant weight/total body weight ratio was used to calculate the hepatic regenerative index. Rats treated with CeO_2_NPs showed a significant 11% increase in the hepatic regenerative index, compared with vehicle-treated rats (*p *< 0.05) (Fig. 2b). The beneficial effect of CeO_2_NPs on liver regeneration was also accompanied by lower blood levels of alanine aminotransferase (ALT), aspartate aminotransferase (AST) and the enzyme lactate dehydrogenase (LDH) after 3 h post-PHx and compared with the vehicle group (Fig. 2c).

To further investigate the cause through which CeO_2_NPs improves liver regeneration, we assessed the expression of the cell proliferation marker Ki67 in liver sections of rats treated with nanoparticles. Rats receiving vehicle or CeO_2_NPs showed absence of hepatocellular proliferation in resting livers (t = 0 h) (Fig. 2d). However after PHx, the remnant liver from rats treated with CeO_2_NPs showed a significant increase in Ki67^+^-hepatocytes at day 1 (*p < 0.05, *t *= 24 h) that reached a maximum at t = 48 h after partial hepatectomy, compared with vehicle (44.6 ± 4.5% vs. 38.5 ± 6.7% Ki67^+^ cells, *p < 0.05, *t *= 48 h); returning to nearly basal levels after 7 days (Fig. 2d). Therefore, improvement of liver regeneration caused by the CeO_2_NPs treatment is associated with enhanced hepatocyte proliferation.

### CeO_2_NPs treated rats showed decreased liver damage and increased hepatocellular proliferation after acetaminophen-induced liver injury

**Fig. 3:**
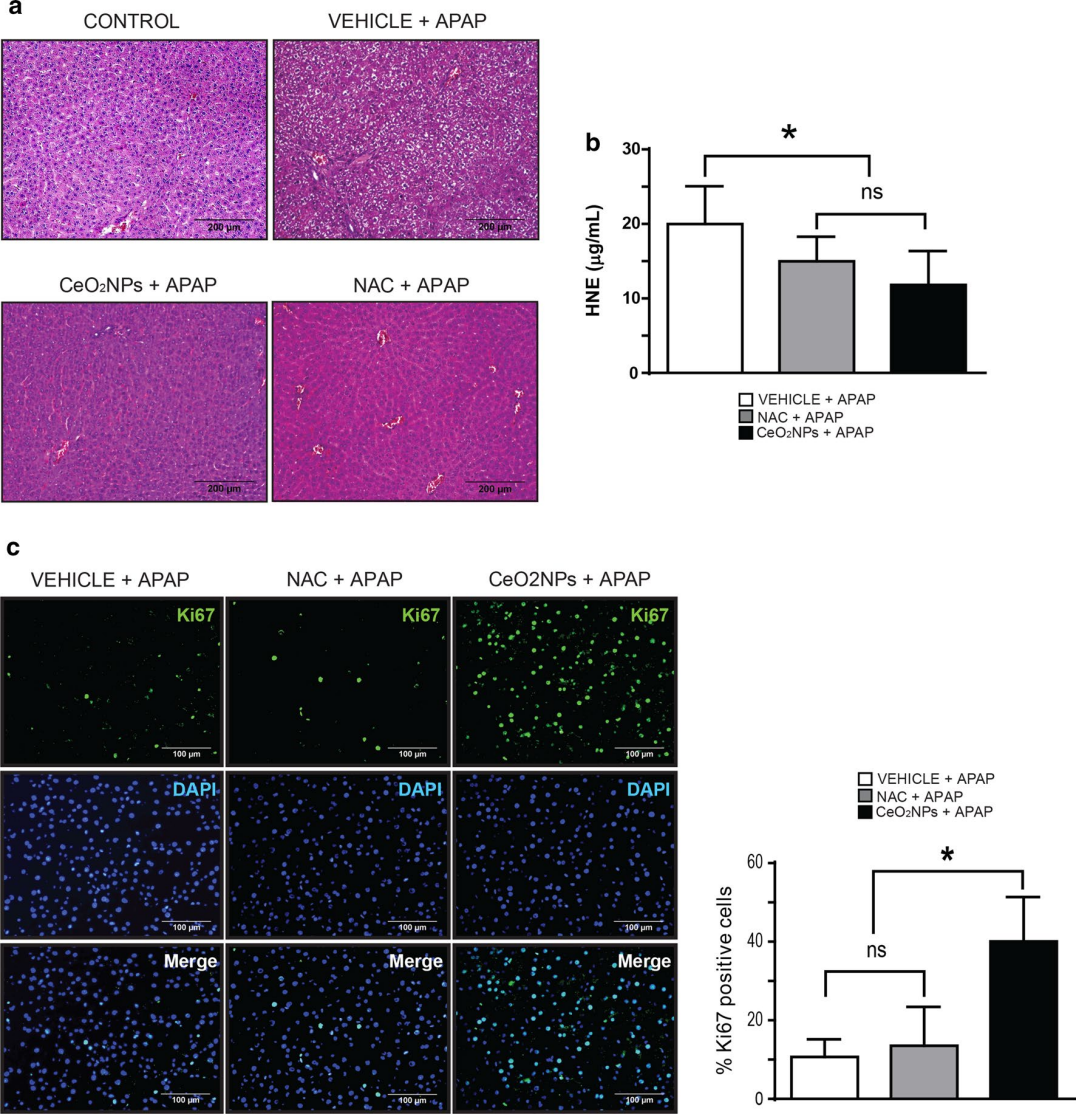
CeO_2_NPs treatment reduces histological damage and increases cell proliferation after APAP-induced injury. **a** Hematoxylin-eosin stained liver sections (n = 13). After vehicle or CeO_2_NPs treatments, rats received 1 g/kg APAP and were sacrificed after 48 h (vehicle + APAP and CeO_2_NPs + APAP, respectively). Another group was treated with 300 mg/kg NAC 1 h after APAP (NAC + APAP). Also, healthy non-treated rats were included as experimental controls (upper left panel), ×100. **b** Quantification of HNE in liver from vehicle + APAP, NAC + APAP and CeO_2_NPs + APAP groups (n = 13; mean ± SEM; *p < 0.05). **c** Immunostaining for Ki-67 in liver of rats treated with vehicle + APAP, NAC + APAP and CeO_2_NPs + APAP. Ki-67 (green) and DAPI (blue), ×200. On the right, quantification of Ki-67 positive cells (n = 13; mean ± SEM; *p < 0.05)

The previous results show us the role played by CeO_2_NPs on liver regeneration in the context of basal levels of ROS. Next we assessed whether CeO_2_NPs is equally effective after ROS induction caused by APAP toxicity. In this experimental model, excessive oxidative stress plays a major role in APAP hepatotoxicity [40]. The injury induced after 48 h of APAP administration was assessed in three experimental groups: rats previously treated with CeO_2_NPs, rats receiving vehicle and rats receiving a simultaneous treatment with NAC, which is the accepted clinical treatment for APAP overdose. Hematoxylin–eosin staining of livers from rats belonging to the vehicle experimental group showed that APAP administration induced severe liver injury characterized by massive necrosis, hepatocyte vacuolation and vascular congestion. Liver injury was significantly reduced in the NAC and the CeO_2_NPs-treated groups, although vascular congestion was still present in both groups despite the NAC and the CeO_2_NPs treatments (Fig. 3a). As expected, APAP treatment induced a significant increase in hepatic ROS that was quantified by measuring 4-hydroxynonenal in the liver tissue (HNE), a widely accepted marker of lipid peroxydation and oxidative damage. Interestingly, both NAC (Fig. 3b second bar in the graph) and CeO_2_NPs (Fig. 3b third bar in the graph) are equally effective as antioxidant treatments for treating APAP-induced oxidative stress when compared with the vehicle condition (Fig. 3b first bar in the graph). Despite sharing beneficial antioxidant properties, NAC and CeO_2_NPs treatments differed in their capability of stimulating hepatocellular proliferation. The liver from rats treated with CeO_2_NPs showed an extensive positivity for Ki67, compared with vehicle and NAC treated rats at day 2 after APAP injection (40.08 ± 5.04%, 10.70 ± 1.69% and 13.53 ± 3.13% Ki67^+^ cells, respectively; *p *< 0.05) (Fig. 3c).

We performed additional experiments to characterize better the liver injury induced by APAP and the degree of recovery of the different experimental groups. To this end, we quantified the serum concentration of transaminases (ALT, AST), glucose and albumin from CeO_2_NPs-treated and vehicle-treated rats at t = 0 h, t = 48 h, and t = 96 h. We found that the concentration of transaminases was significantly increased to a similar extent in the vehicle and the CeO_2_NPs-treated groups 2 days after APAP administration (t = 48 h compared with t = 0 h). However, CeO_2_NPs treatment was associated with a significant decline in serum ALT, reaching basal levels at 96 h after the APAP injection; compared with the vehicle group (Additional file 1: Figure S2). This recovery phase marker, which is a more specific marker of liver injury than AST, points to a more efficient recovery of the CeO_2_NPs-treated liver after drug-induced liver damage.

### CeO_2_NPs treatment stimulates cell cycle progression in HepG2 cells and NFκB activation in vitro and in vivo

**Fig. 4:**
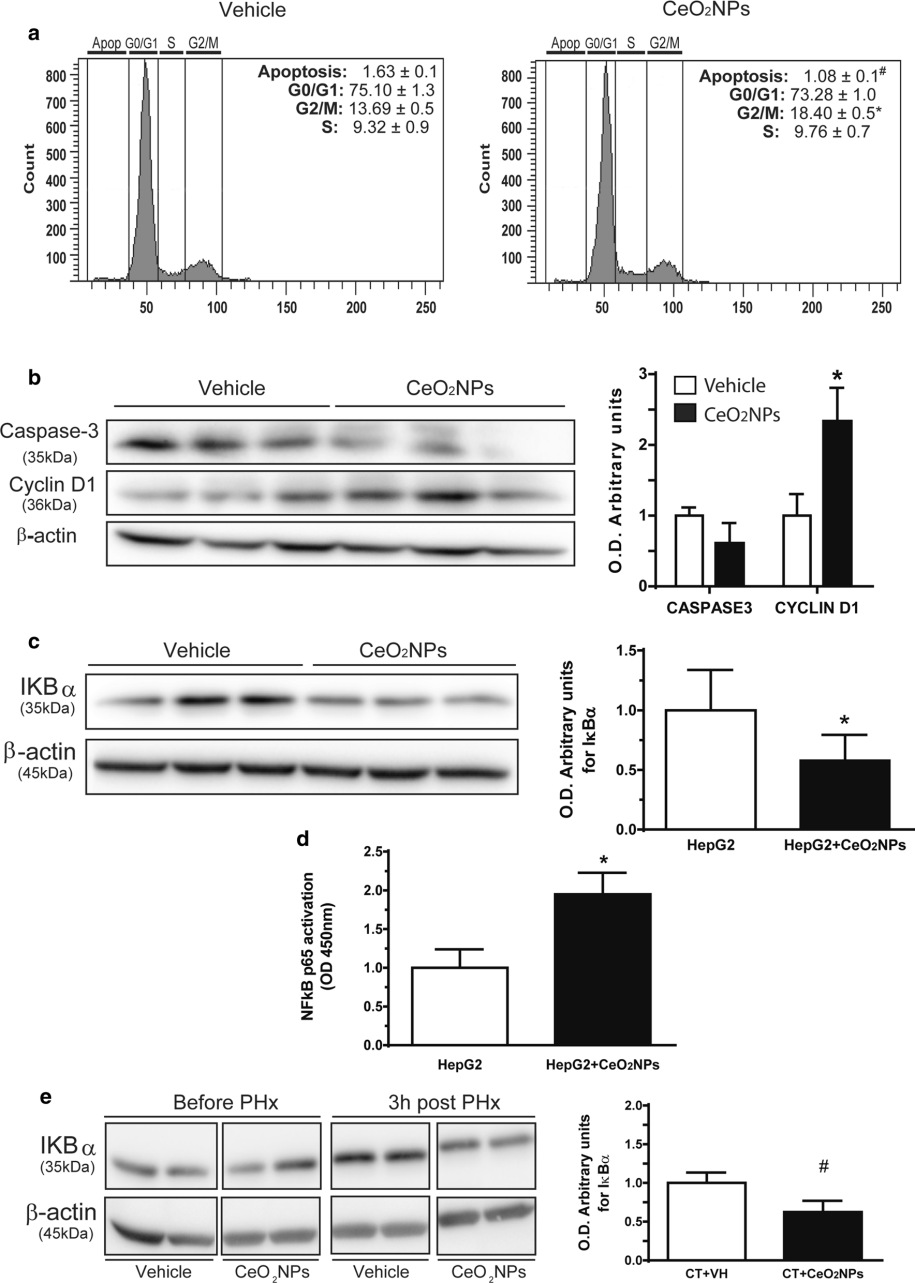
CeO_2_NPs stimulates cell cycle progression and NF-κB activation. **a** Flow cytometry of HepG2 showing cell cycle profiles from propidium iodide DNA staining after vehicle or CeO_2_NPs treatment (n = 5; ^*#*^*p *< 0.01 and *p < 0.05). **b** Western blot for activated caspase 3 and cyclin D1 abundance from HepG2 incubated with vehicle or CeO_2_NPs. β-actin was used as loading control (mean ± SEM; n = 5; *p < 0.05 versus vehicle in the same experimental condition). O.D.: optical density. **c** Western blot for IκBα abundance from HepG2 (mean ± SEM; n = 5; *p < 0.05). **d** Transcription factor immunosorbent assay for NF-κB (p65) activity in HepG2 (n = 5, *p < 0.05). **e** Western blot for IκBα abundance in the liver vehicle and CeO_2_NPs-treated rats before and 3 h post-PHx (mean ± SEM; n = 5; ^#^*p *< 0.01)

To ensure the generalization of the finding that CeO_2_NPs improves hepatocyte proliferation in vivo after PHx and APAP-induced toxicity in rats, we characterized the dynamics of the cell cycle in the human hepatocyte cell line HepG2. As shown in Fig. 4a, CeO_2_NPs treatment significantly decreased the percentage of HepG2 cells that underwent apoptosis following 48 h of serum starvation, as detected by propidium iodide staining and flow cytometry. Moreover, CeO_2_NPs treatment was associated with a higher percentage of HepG2 cells that were in the G2/M phase of the cell cycle, reflecting the stimulatory role played by CeO_2_NPs on cell cycle progression. We further validate these observations in the HepG2 cells by quantifying active caspase-3 and cyclin D1 (markers of apoptosis and cell cycle progression, respectively). CeO_2_NPs treatment of HepG2 cells reduced the amount of activated caspase-3 and increased the levels of cyclin D1 following 48 h of serum starvation (Fig. 4b).

The NF-κB/Rel family of transcription factors (NFκB) is a central hub of signaling pathways that regulates apoptosis and cell proliferation. In addition, the activity NFκB is modulated by ROS [41–43]. These confluent evidences let us to assess whether the activity of NF-κB is affected by the CeO_2_NPs treatment in vitro and in vivo. In HepG2 cells treated with CeO_2_NPs, the abundance of IκBα (that function as an inhibitor of NFκB) significantly decreased when compared to vehicle-treated HepG2 cells (p < 0.05) (Fig. 4c). Next, the transcriptional activity of NF-κB was assessed by detecting specific p65 DNA binding in nuclear extracts from CeO_2_NPs treated and non-treated HepG2 cells. As shown in Fig. 4d, CeO_2_NPs treated cells showed increased p65 transcriptional activity compared with non-treated HepG2 cells (p < 0.05). We next investigated whether the described stimulation of NF-κB activity by CeO_2_NPs was also detected in regenerative livers in vivo. Before PHx, there were no differences in the expression of IκBα in the livers from vehicle or CeO_2_NPs treated rats. However, and in agreement with the results obtained in HepG2, the abundance of IκBα was significantly lower in the livers from CeO_2_NPs treated rats 3 h after PHx, compared with the group of rats receiving vehicle (p < 0.01) (Fig. 4e).

## Discussion

We previously described that CeO_2_NPs showed hepatoprotective activity in vivo against fibrosis [31]. Another study also demonstrated the beneficial effects of these nanoparticles against hepatic oxidative damage caused by the pyrrolizidine alkaloid monocrotaline, which causes oxidative vascular damage in the liver [44]. Considering this hepatoprotective role of CeO_2_NPs, we further investigated whether these nanoparticles might also stimulate other hepatoprotective functions, such as liver regeneration. In the present study we describe for the first time that CeO_2_NPs stimulate liver regeneration in the rat experimental models of partial hepatectomy and DILI caused by acetaminophen overdose. Consistently with this observation, we demonstrated that CeO_2_NPs treatment decreases liver damage after PHx and APAP-induced liver injury. The treatment with CeO_2_NPs also leads to the reduction of oxidative stress markers and stimulates the cell cycle progression in hepatocytes and the activation of the transcription factor NF-κB in vitro and in vivo.

It is known that excess of oxidative stress inhibits efficient regeneration after partial hepatectomy or acute liver failure caused by DILI. For example, the genetic deficiency of Nrf2 (a regulator of the cellular antioxidant response) and UCP2 (an uncoupling protein of the electron transport chain) cause enhanced oxidative stress and impaired liver regeneration in partially hepatectomized mice [43]. Moreover, oxidative cellular damage in mitochondria and DNA is associated with deficient liver regeneration after acetaminophen toxicity [45]. Therefore, several strategies have been investigated by others to diminish ROS in the context of liver regeneration in preclinical models. For example, thymoquinone, an essential oil with free radical scavenging capacity, protects rat liver against ischemia/reperfusion injury [46] and prevents liver injury in hepatectomized mice under I/R by blunting oxidative stress, mitochondrial damage, endoplasmic reticulum stress and apoptosis [47]. Exogenous administration of GSH or resveratrol significantly decreased oxidative stress and protect against APAP-induced liver injury [48, 49]. Similarly, pharmacological inhibition of JNK activity, which enhances mitochondrial-derived ROS production upon APAP toxicity, reduced liver damage [50]. Some drugs approved for its clinical use in different pathologies have also been tested pre-clinically for APAP hepatotoxicity. Both methylene blue and metformin reduced mitochondrial oxidant stress [51] and protected against APAP liver injury [52].

Our study has potential limitations regarding the APAP-induced toxicity model in rats. It has been shown that rats are more resistant to acetaminophen damage than mice [53]. Despite this observation, the presence of oxidative stress in the APAP-induced toxicity model in rats has broadly been demonstrated [54–56], regardless of the severity of the injury between species. Therefore, this experimental model in rats is still valid to test the antioxidant properties of CeO_2_NPs treatment on liver regeneration after drug-induced injury. Regarding the metabolism of APAP in the rat liver, we found in our study that the concentration of transaminases was significantly increased to a similar extent in the vehicle and the CeO_2_NPs-treated groups 2 days after APAP administration. This result confirmed that there is significant toxicity induced by APAP overdose in our experimental rat model and that the CeO_2_NPs pretreatment does not modify the metabolism of APAP in the liver. Nevertheless, the quantification of an intermediary metabolite of APAP (i.e., *N*-acetyl-*p*-benzoquinone imine) would have supported more robustly this conclusion.

Our study presents an innovative treatment for diminish ROS in the context of liver regeneration using nanoparticles. Some of the advantages of using CeO_2_ nanomaterials over the traditional anti-oxidative drugs are: (1) minimal toxicity in cumulative doses [57, 58], (2) the multi-enzyme mimetic activities of CeO_2_NPs, which targets several sources of ROS generation and (3) the continuous regeneration of the CeO_2_NPs catalytic activity, which avoids the exhaustion of its anti-oxidative properties [58]. Among these theoretical benefits over traditional drugs, we also observed superior therapeutic performance compared with the gold standard treatment used for acetaminophen toxicity in patients, NAC. In our study, CeO_2_NPs treatment is equally effective reducing oxidative stress and tissue damage in rats receiving APAP overdose compared with NAC. However, while NAC did not affect hepatocyte proliferation in injured livers, CeO_2_NPs robustly increased cell proliferation in vivo and in vitro. In addition, we observed a significant decrease in the percentage of CeO_2_NPs-treated HepG2 cells that underwent apoptosis following 48 h of serum starvation, suggesting that the anti-apoptotic effect associated to the nanoceria treatment may also contribute to enhance liver regeneration.

NF-κB plays a major role in liver homeostasis and liver regeneration. For instance, NF-κB (p65) knockout mice show embryo lethality and display massive hepatocyte apoptosis [59]. In addition, the transduction of rat livers with different forms of IκB, an inhibitor of NF-κB activity, before PHx was associated with impaired liver regeneration [60]. Furthermore, the inactivation of NF-κB in Kupffer cells as well as in hepatocytes impaired the regenerative process after PHx [61]. Here, we demonstrated that the treatment with CeO_2_NPs lead to the activation of the transcription factor NF-κB in vitro and in vivo. Our results are not sufficient to establish a robust link between NF-κB activation and the beneficial effects of CeO_2_NPs treatment on liver regeneration yet. However, the agreement between our findings and the studies mentioned above make this relationship plausible.

It has been described that oxidative stress can inhibit the transcriptional activity of NFκB through different mechanisms including direct interaction with NF-κB, inactivation of IKK (an IκB repressor), and alternative inhibitory phosphorylation of IκBα [41]. Our in vitro and in vivo results showed that CeO_2_NPs are associated with a significant decrease in the intracellular IκBα content, which is translated in the activation of NF-κB. Therefore, our findings suggest that the anti-oxidant property of CeO_2_NPs is responsible for the increase in NF-κB activation. However, our results are in disagreement with other studies which have shown that CeO_2_NPs treatment inhibits NF-κB in vivo mostly in the pathological context of sepsis [62–64]. One of the reasons that may explain the seemingly contradictory findings is differences in the CeO_2_NPs stability used in these studies, what has been recently recognized as key in order to employ CeO_2_NPs for medicine [22]. In addition, these studies used higher in vivo doses of CeO_2_NPs compared with ours (from 0.5 mg/kg to 3.5 mg/kg b.w. compared with 0.1 mg/kg b.w.). Our previous dosage standardization experiments [31] indicate that at higher doses CeO_2_NPs shows a hormetic-like dose response characterized by an increasing dose inefficacy in ROS scavenging.

## Conclusions

In summary, drug-induced hepatotoxicity and impaired liver regeneration are major concerns in medical practice as they are leading causes of acute liver failure and liver transplantation. Our results demonstrated that CeO_2_NPs, besides their known antioxidant capacities, can enhance liver regeneration in experimental models that replicate these two clinical scenarios. Hence, CeO_2_NPs treatment may provide avenues to overcome deficient liver regeneration in patients.

## Methods

### Synthesis and characterization of CeO_2_NPs

CeO_2_NPs were synthesized by the chemical precipitation of cerium (III) nitrate hexahydrated (Sigma-Aldrich, St. Louis, MO, USA) in aqueous solution, as reported elsewhere [19]. Controlling the pH of synthesis, small-sized nanoceria can be obtained. Here, we used 4 nm nanoparticles at a concentration of 1 mg/mL. The surface charge of the NPs was characterized by the Z-potential in a Z-sizer (Malvern, Worcestershire, UK) while the crystal size was characterized by High Resolution Transmission Electron Microscopy (HR-TEM) in the Tecnai G2 F20 (FEI, Oregon, USA). Nanocrystalline morphology and composition was analyzed by HR-TEM (Tecnai 200 kV) and XRD (Xpert Pannalytical, MA, USA), and the light interaction by UV–VIS spectroscopy (Shimatzu, Kyoto, Japan). Size distribution was computer analyzed by ImageJ (National Institutes of Health, Bethesda, MD, USA). CeO_2_NPs were stabilized with Tetramethylammonium hydroxide (TMAOH), which was used as vehicle in all the experiments as a control condition, and kept at 4 °C until use. Prior to the animal studies, 15 µL from the nanoparticles solution were dispersed on a copper grid coated with a formvar film. The samples were then let to dry for TEM observation and digital photomicrographs were taken (BioScan Gatan, CA, USA). Free CeO_2_NPs samples available for evaluation if requested.

### Animal experimentation and In vivo CeO_2_NPs treatment

This study was performed in male Wistar rats (Charles-River, Saint Aubin les Elseuf, France). Rats were fed with standard chow diet and housed on a 12 h light/12 h dark cycle. For each experiment of this study, treated rats were administered 0.1 mg/kg CeO_2_NPs dispersed in saline solution as a bolus (500 μL), given intravenously through the tail vain twice a week for 2 weeks. Control groups without treatment and treated with vehicle (TMAOH) were also included in the study. At the end of each experiment rats were euthanized by isoflurane anesthesia overdose.

### CeO_2_NPs tissue quantification

For tissue quantification, at the end of the 2 weeks treatment, rats were euthanized at days 1, 21, 42, and 56 (90 min, 3, 6 and 8 weeks respectively) and their livers, spleens, lungs and kidneys dissected and kept at − 80 °C for further analysis. For CeO_2_NPs quantification, samples were diluted in an aqueous solution of HNO_3_ 2% w/w (Trace Metal Basis; Sigma-Aldrich, St. Louis, MO, USA) and analyzed for cerium concentration by inductively coupled plasma mass spectrometry (ICP-MS, Agilent 7500; Agilent Technologies, California, USA). The quantitative results were obtained by interpolation in a standard curve obtained from a commercial 1000 ppm Ce standard (Sigma-Aldrich, St. Louis, MO, USA).

### Reactive oxygen species quantification

Reactive oxygen species were measured by fluorescence spectrophotometry using 2′,7′-DCF-DA as a probe. For these experiments, HepG2 cells alone or treated with H_2_O_2_ in the presence or absence of CeO_2_NPs were washed with phosphate-buffered saline (PBS) and incubated with 10 μM DCF-DA (Thermo Fisher Scientific, Waltham, MA, USA) in Dulbecco’s modified Eagle medium (DMEM) for 40 min at 37 °C in the dark. The supernatant was collected to measure the extracellular production of ROS, and the intensity of fluorescence was immediately read in a fluorescence spectrophotometer (FLUOstar Optima; BMG Labtech, Ortenberg, Germany) at 485 nm for excitation and at 520 nm for emission.

### In vitro CeO_2_NPs treatment

The human cell line HepG2 (a suitable in vitro model system for the study of polarized human hepatocytes) was obtained from the American Type Culture Collection (ATCC; Manassas, VA, USA). HepG2 cells were cultured and maintained in DMEM containing 10% FBS, 100 U/mL penicillin and 100 μg/mL streptomycin at 37 °C in a humidified atmosphere containing 5% CO_2_. Cells were serum-starved and treated with 0.1 mg/mL CeO_2_NPs, or vehicle as a control condition, for 48 h.

### Measurement of NF-κB activation

NF-κB activity in HepG2 cells was measured using a NF-κB Transcription Factor Assay Kit (specific for p65), following the manufacturer’s instructions (Cayman Chemical, Ann Arbor, MI, USA). To this end, nuclear extracts from HepG2 were incubated with double stranded DNA immobilized in a 96-well plate that contained the κB response motif site. The complex p65-DNA was detected by enzyme-linked immunosorbent assay using specific primary antibody directed against p65 and a secondary antibody conjugated to HRP. The results were quantified by measuring absorbance at 450 nm in a FLUOstar Optima (BMG Labtech, Ortenberg, Germany).

### Statistical analysis

Quantitative data were analyzed using GraphPad Prism, version 6 (GraphPad Software, Inc., San Diego, CA, USA) and public libraries from The Comprehensive R Archive Network (CRAN; http://CRAN.R-project.org) rooted in the open source statistical computing environment R, version 3.4 (http://www.R-project.org/). The statistical analysis of the results was performed using unpaired Student’s t-tests and ANOVA models (with Tukey’s post hoc test) with normally distributed data. For other type of data, the Mann–Whitney U-test and the Kruskal–Wallis tests (with Dunn post hoc test) were used. Results are expressed as mean ± sem and considered significant at a p value lower than 0.05.

Additional Materials and Methods are shown in Additional file 1. Free CeO_2_NPs samples available for evaluation if requested.

## Supplementary information

**Additional file 1.** Additional materials and methods, additional figures and references.

## Data Availability

All data generated or analyzed during this study is available from the corresponding author on reasonable request.

## Abbreviations

CeO_2_NPs: cerium oxide nanoparticles
PHx: partial hepatectomy
APAP: acetaminophen
NAC: *N*-acetyl-cysteine
DILI: drug-induced liver injury
ROS: reactive oxygen species
DMEM: Dulbecco’s modified Eagle medium
PBS: phosphate-buffered saline
DCF: dichlorofluorescein
HNE: 4-hydroxynonenal
PI: propidium iodide
ALT: alanine aminotransferase
AST: aspartate aminotransferase
LDH: lactate dehydrogenase
