# Supplementary material for: Cerium oxide nanoparticles improve liver regeneration after acetaminophen-induced liver injury and partial hepatectomy in rats
Source: J Nanobiotechnology. 2019 Oct 31;17:112. doi: 10.1186/s12951-019-0544-5 (PMC6822381; doi:10.1186/s12951-019-0544-5)
Supplement: Supplementary file 1 — Additional file 1. Additional materials and methods, additional figures and references. [file 12951_2019_544_MOESM1_ESM.docx]

**ADDITIONAL INFORMATION**

**Cerium oxide nanoparticles improve liver regeneration after acetaminophen-induced liver injury and partial hepatectomy in rats**

Bernat Córdoba-Jover^1,&^, Altamira Arce-Cerezo^1,&^, Jordi Ribera^1^, Montse Pauta^1^, Denise Oró^1^, Gregori Casals^1,2^, Guillermo Fernández-Varo^1^, Eudald Casals^3,4,6^, Victor Puntes^3,4,5^, Wladimiro Jiménez^1,7^ and Manuel Morales-Ruiz^1,2,7*^.

^&^Both authors contributed equally to this study

*Corresponding authors:

-Manuel Morales-Ruiz, Ph.D., Biochemistry and Molecular Genetics Department, Hospital Clinic, 170 Villarroel St. Barcelona, 08036, Spain. Tel: +34-932275466, Fax: +34-932275697, e-mail: [morales@clinic.cat](mailto:morales@clinic.cat)

**Table of content:**

1. Additional Materials and methods

2. Additional Figure and Legend

3. References

**Additional Materials and methods**

***Experimental model of partial hepatectomy.***

All the surgical procedures performed in rats were made under isoflurane anesthesia (Sigma-Aldrich, St. Louis, MO). Partial hepatectomy (PHx) was performed according to the technique described by Higgins and Anderson[1]. The abdomen was opened via a midline incision and the median and left lobes were removed. After PHx, control and treated rats were euthanized at different time points. The regenerating bottom right lobe was snap-frozen into liquid nitrogen and the upper right lobe was fixed in 4% paraformaldehyde (PFA) at 4°C, cryoprotected overnight in 30% sucrose solution, and embedded in OCT medium (Tissue-Tek® O.C.T™ Compound, SAKURA) and frozen for future processing. The percentage of liver regeneration was calculated following the formula: weight of non-removed lobes/total body weight of rats, as previously described [1].

***Acetaminophen (APAP) toxicity and N-acetyl-cysteine treatment.***

After vehicle administration or CeO_2_NPs treatment, rats were administered 1g/kg APAP (Acetaminophen BioXtra, ≥99.0%, Sigma Aldrich, St. Louis, MO) intraperitoneally and euthanized at 0h, 48h and 96h to obtain liver and serum samples for further analysis. As a therapeutic control group, thirteen rats were treated with 300mg/kg N-acetyl-cysteine (NAC) (*N*-Acetyl-L-cysteine, Sigma Grade, ≥99% [TLC], Sigma Aldrich, St. Louis, MO) intraperitoneally 1h after APAP administration. Liver left lobes were removed from these rats and dissected into pieces for: 1/ fixation in 4% PFA at 4ºC, cryoprotection in 30% sucrose solution overnight and inclusion in OCT medium (Tissue-Tek® O.C.T™ Compound, SAKURA); 2/ fixation in 10% formaldehyde and paraffin inclusion and 3/ storage in liquid nitrogen for protein extraction. APAP-induced liver injury was microscopically evaluated by performing hematoxylin-eosin staining (Hematoxylin Solution, Gill No. 3, Sigma Aldrich, St. Louis, MO) on liver sections included in paraffin. All the histology images were taken using a light microscope coupled with a digital image acquisition system (Nikon Eclipse E600, Kawasaki, Kanagawa, Japan). In addition, the presence of oxidative stress in the livers of the APAP-treated animals was determined by measuring the end product of lipid peroxidation 4-hydroxynonenal (HNE), a well known marker for oxidative damage, using OxiSelect™ HNE Adduct Competitive ELISA kit (Cell Biolabs Inc., San Diego, CA), following manufacturer’s instructions.

***Immunofluorescence.***

For both models, PHx and APAP, frozen sections of 8-μm were rehydrated, blocked with 5% normal goat serum and incubated with mouse anti-ki67 (1:100, Abcam, ab16667). Controls without primary antibodies were revealed with Alexa-488 goat-anti-rabbit IgG (1:500, Thermo Fisher Scientific, Waltham, MA, USA). Next, slides were mounted with mounting medium containing DAPI (VECTASHIELD®, Vector Laboratories Inc., Burlingame, CA, USA). Tissue slides were visualized with a fluorescence microscope (Nikon Eclipse E600, Kawasaki, Kanagawa, Japan). Ki67 positive cells were counted using Image J (version 1.37, National Institutes of Health, Bethesda, MD).

***Western blot analysis.***

Tissue lysates were prepared in a lysis buffer (Tris–HCl 20 mM pH 7.4 containing 1% Triton X-100, 0.1% SDS, 50 mM NaCl, 2.5 mM EDTA, 1 mM Na_4_P_2_O_7_ 10H_2_O, 20 mM NaF, 1 mM Na_3_VO_4_, 2 mM Pefabloc and Complete® from Roche, Basel, Switzerland). Proteins were separated on a 10% SDS-polyacrylamide gel (Mini Protean III, BioRad, Richmond, CA, USA) and transferred for 1 hour at 4ºC to 0,2 µm nitrocellulose transfer membranes (BioRad, Richmond, CA, USA). Membranes were incubated at 4ºC overnight with the following antibodies; rabbit anti-IKB alpha (1:1000, ab32518, abcam, Cambridge, UK), rabbit anti-Cyclin D1 (1:1000, #2922, Cell Signaling, Danvers MA, USA), rabbit anti-active Caspase-3 antibody (1:1000, ab32042, abcam, Cambridge, UK) and rabbit anti β-actin (13E5 HRP conjugate, 1:1000, #4970, Cell Signaling, Danvers MA, USA). Membranes were incubated with a donkey ECL™-anti-rabbit IgG horseradish peroxidase-conjugated secondary antibody at 1:5000 dilution (GE Healthcare, Chicago, IL, USA) for 1 hour at room temperature. Bands were visualized using Luminata^TM^ Forte Western HRP Substrate (Merck Millipore, Burlington, MA, USA) and ImageQuant^TM^ LAS 4000 (GE Healthcare, Chicago, IL, USA). Densitometry analysis of membranes was performed using Image J (version 1.37, National Institutes of Health, Bethesda, MD).

***Cell cycle analysis.***

HepG2 cells were washed twice with ice-cold PBS, fixed in 70% cold ethanol, treated with 100 μg/mL ribonuclease A (Roche, Basel, Switzerland) and labeled with 50 μg/mL propidium iodide (PI) for at least 4h at 4°C. Next, cells were analyzed by flow cytometry (FACScalibur, Becton-Dickinson) using selective gating to exclude the doublets of cells and quantified using the MODFIT software (Verity Software House, Inc.).

***Biochemical assays****.*

At the end of the treatment, rats were euthanized, serum was obtained by blood centrifugation and biochemical parameters were assayed using an automatic chemistry analyzer (BS-200E, Mindray Medical International LTD, Shenzhen, China). In these samples, aspartate aminotransferase (AST), alanine transaminase (ALT), glucose, albumin, and lactate dehydrogenase (LDH) were determined.

**Additional Figures and Legends**

**
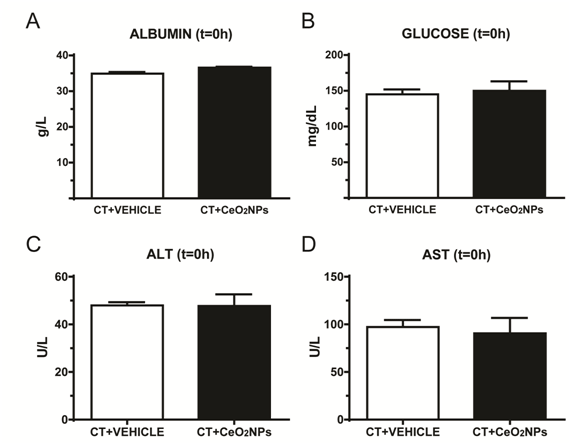
**

**Figure S1. Measurement of serum laboratory parameters in rats treated with vehicle and CeO_2_NPs before PHx or APAP *i.p.* injection.** Serum concentration of albumin, glucose, ALT and AST in rats treated with vehicle (white bars, n=8) and CeO_2_NPs (black bars, n=8) at t=0 hours before PHx or APAP treatment. No significant differences were found between groups in any of the biochemical parameters evaluated.


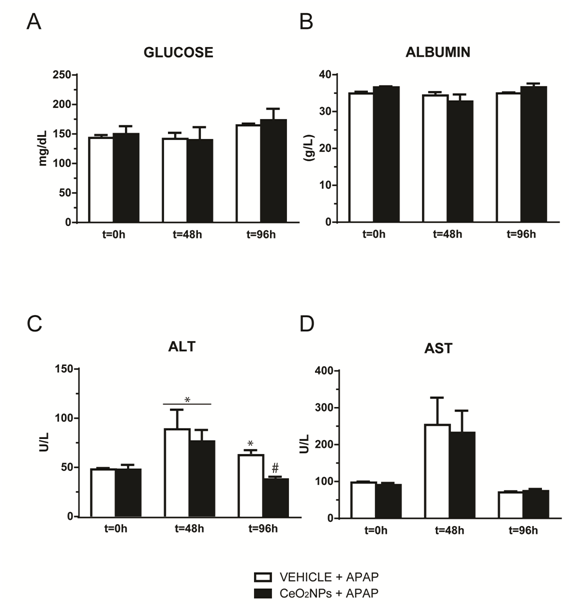


**Figure S2. Measurement of serum laboratory parameters in rats treated with vehicle and CeO_2_NPs after APAP i.p. injection.** Serum concentration of glucose, albumin, ALT and AST in rats treated with vehicle (white bars, n=8) and CeO_2_NPs (black bars, n=8) at t=0h, t=48h and t=96h after APAP *i.p*. injection *(*p<0.01* compared with t=0 and CeO_2_NPs treatment at t=96h; *^#^p<0.05* compared with vehicle at the same time points).

**References**

1. Higgins GM. Anderson. RM. Experimental pathology of the liver: Restoration of the liver of the white rat following partial surgical removal. Arch Pathol. 1931;12:186–202.
